# Chimaphila for Diabetes

**Published:** 1912-01

**Authors:** 


CHIMAPHILA FOR DIABETES.—S. G. Saules reports the disappearance of sugar from the urine of a patient who had suffered from diabetes for eight years in three weeks after beginning treatment with chimaphila. Capsicum and grindelia robusta had been found efficacious in reducing the amount of sugar.—*Ellingwood's Therapeutist.*

